# Rapid development of Philadelphia chromosome-negative AML in a CML patient with sustained major molecular response to tyrosine kinase inhibitor therapy

**DOI:** 10.1016/j.lrr.2025.100536

**Published:** 2025-08-21

**Authors:** Huan Liu, Yunxia Sun, Liangliang Li, Yurong Zhang, Yaxiong Zhou, Pengyun Zeng, Lingling Yue

**Affiliations:** a Department of Hematology, The Second Hospital & Clinical Medical School, Lanzhou University, No. 82, Cuyingmen, Lanzhou, Gansu Province 730030, China; b Department of Hematology, The First People's Hospital of Lanzhou, China; c Department of Cardiac Surgery, The Second Hospital & Clinical Medical School, Lanzhou University, China

**Keywords:** Chronic myeloid leukemia (CML), Acute myeloid leukemia (AML), Complete cytogenetic response (CCyR), Major molecular response (MMR), Clonal chromosomal abnormalities in Philadelphia chromosome-negative (CCA/Ph-)

## Abstract

The use of TKIs has significantly improved the prognosis of CML. However, a small subset of patients still experience poor outcomes. We present a rare case of Ph-AML following a diagnosis of CML. The patient achieved CCyR and MMR after 4 months of nilotinib therapy, with sustained deep remission for 3 years. Unexpectedly, the disease developed rapidly to AML. Further investigations revealed the emergence of CCA/Ph- and gene mutations. We retrospectively analyzed previous CML patients with *BCR::ABL1* and Ph-negative status in blast crisis from our database and conducted a comprehensive review of the relevant literature.

## 1 Introduction

CML is a malignant hematopoietic disorder characterized by a reciprocal chromosomal translocation, t(9;22)(q34;q11.2), which results in the formation of the *BCR::ABL1* fusion gene. This gene produces a constitutively active *BCR::ABL1* tyrosine kinase, driving uncontrolled cell proliferation and clonal expansion. The advent of tyrosine kinase inhibitors (TKIs) has revolutionized the treatment of CML, transforming it into a manageable chronic condition and significantly improving patient outcomes [1]. However, a subset of patients continues to face poor prognosis despite treatment.

Clonal chromosomal abnormalities (CCAs) in patients with CML can occur in Philadelphia chromosome-negative (Ph-) cells, referred to as CCA/Ph- [2]. The reported common clonal chromosomal abnormalities in CCA/Ph- cells include + 8, - 7 and -Y [3,4]. Several studies have demonstrated that CCA/Ph- is relatively rare and typically transient. Development to AML in such cases is extremely rare. We present a rare case of CCA/Ph- AML that demonstrated a deep remission to nilotinib and review similar cases previously reported.

## 2 Case report

A 75-year-old female was admitted to our hospital in January 2021 due to abdominal distension. Blood tests revealed a white blood cell count of 369.26 × 10⁹/L, and giant spleen. Reverse transcription polymerase chain reaction (RT-PCR) detected the BCR/ABL p210 fusion gene, and karyotype analysis revealed 46,XX,t(9;22) (q34;q11). The patient was diagnosed with CML and started on nilotinib 300 mg orally twice daily. The patient achieved CCyR and MMR 4 months after diagnosis. Regular monitoring confirmed continued CCyR and MMR during 3 years of nilotinib therapy. However, on August 31, 2024, the patient was readmitted our hospital. Routine blood tests showed a white blood cell count of 7.46 × 10⁹/L, hemoglobin 59 g/L, and platelets 9 × 10⁹/L. Bone marrow smear showed 36 % blasts. Immunophenotyping revealed AML phenotype. Interestingly, correlation analysis revealed the absence of *BCR::ABL1* fusion gene variants, kinase domain mutations, and leukemia-associated fusion genes. New chromosomal abnormalities were observed, specifically 45,XX,t(3;17)(q26;q22),−7[10]. Whole-exome sequencing of 256 hematological tumor-related genes identified mutations in KRAS (4.14 %), NF1 (39.76 %), PTPN11 (26.62 %), and RUNX1 (24.49 %). The patient was started on the VA chemotherapy regimen, consisting of venetoclaxand and azacitidine. Six days later, the patient’s platelet count decreased to 1 × 10⁹/L, and she died from a cerebral hemorrhage.

## 3 Discussion

The patient achieved CCyR and MMR during TKI therapy but later experienced rapid development to AML. Notably, no additional cytogenetic abnormalities were detected during CCyR and MMR . However, new chromosomal abnormalities and gene mutations emerged upon development to AML. We conducted a comprehensive review of the relevant literature. To date, only 39 cases of CML with Ph- development to AML have been reported globally. According to our analysis, of the 39 case cohort, 2.5 % (1/39) had undocumented gender. The 38 documented cases exhibited a male predominance, the male-to-female ratio was approximately 1.53:1 (23/15). The median age was 57 years (range 22–81 years). Of these, 7 cases developed to AML before MDS, and 1 case developed to acute promyelocytic leukemia (APL). The most common chromosomal abnormality in CML development to AML was −7, observed in 35.8 % (14/39) of cases, followed by +8, seen in 17.9 % (7/39). Additional genetic mutations were identified in 12 of the 39 patients who developed to AML, with mutations in genes such as NPM1, FLT3-ITD, FLT3-D835, TET2, DNMT3A, IDH1, RUNX1, SRSF2, CEBPA, WT1, ATRX, CSMD1, IKZF1, LRP1B, PRPF8, NR2E1, ATM, KRAS, PHF6, MYC, and MLL amplifications. Among these, 12.8 % (5/39) had the NPM1 gene mutatio, followed by FLT3-ITD mutation, 5.1 % (2/39).

Craig Kovitz et al. [5] demonstrated that the probability of *BCR::ABL1* negative CML developing to acceleration or blast crisis is extremely low, approximately 0.1 %, with the proportion of chromosomal abnormalities in Ph-negative cases being less than 2%. Some studies [6] suggest that CCA/Ph- in CML are transient, while a few patients exhibit persistent CCA/Ph- for extended periods. Furthermore, these researchers argued that CCA/Ph- does not significantly affect the long-term prognosis of CML. For instance, in the study by Wasilewska et al. [6], two cases of CCA/Ph- were involved −7 and +8, both of which persisted for 8 and 9 years, respectively. However, in rare cases, CCA/Ph- may indicate a potential transformation to MDS or AML [5]. H. Ni et al. [3] studied 1412 CML patients, among whom 24 cases (1.6 %) exhibited CCA/Ph- during treatment with TKIs. The most common chromosomal abnormalities in these cases were +8, −7, 7q- and 20q-. A single-center study [7] conducted in China surveyed 2115 CML patients over 17 years, finding that 63 cases (2.9 %) had CCA/Ph-, of which only 7 cases (0.3 %) developed to MDS/AML. This study found that −7 and +8 were more frequently observed in the MDS/AML cohort, with +8 being more predominant in the stable disease group. These findings are consistent with our own statistical data and the research conducted by H. Ni.

In our case, the patient was diagnosed with CCA/Ph- and exhibited chromosomal abnormalities, including −7. Deininger et al [8] proposed that no specific treatment is necessary when CCA/Ph- is present without disease development. Wasilewska [6] demonstrated that CML patients who achieve CCyR or MMR may persist with CCA/Ph-, and it remains uncertain whethe TKI therapy can be discontinued in such patients. In contrast, Karimata et al. [9] argued that for patients with −7, proactive interventions, such as allogeneic hematopoietic stem cell transplantation, should be considered before disease development occurs. Other studies have shown that CML patients with −7 have a higher likelihood of developing to MDS or AML compared to those with other CCA/Ph- [10]. Our case and literature review suggest that CCA/Ph- is a dynamic and complex phenomenon that requires close monitoring during CML treatment. Active intervention should be considered when chromosomal abnormalities involving chromosome 7 emerge.

The development of CML to AML accompanied by CCA/Ph- represents a rare clinical phenomenon, with correspondingly scarce documentation of associated genetic mutations in existing literature. Our statistical analysis, encompassing 39 cases to date, identified 12 cases with gene mutations, most commonly involving NPM1 and FLT3-ITD. In our case, no gene mutations were detected at the time of initial diagnosis or during maintenance treatment for CML. However, upon development to AML, mutations in KRAS, NF1, PTPN11, and RUNX1 were identified. Currently, the number of cases of CML with CCA/Ph- developing to AML and associated with gene mutations remains limited, with all instances reported as isolated cases. Large-scale studies are lacking to confirm whether these gene mutations play a critical role in disease development. Further investigation is therefore warranted.

## 4 Conclusion

In this case, the patient with CML rapidly development to AML despite achieving long-term CCyR and MMR after nilotinib treatment. The development of CML to MDS or AML following CCyR and MMR with TKI therapy occurs in a very small number of patients. Based on our case and a review of the literature, we recommend heightened vigilance and proactive treatment when −7 is detected in CCA/Ph-. Moreover, these cases underscores the importance of continuous monitoring of cytogenetics and gene sequencing, even in CML patients with favorable treatment responses, as such monitoring could facilitate the early detection of risk factors that may contribute to disease development. Further research is needed to determine whether CCA/Ph- and acquired gene mutations contribute to suboptimal TKI treatment response, TKI resistance, or subsequent disease development.

## Data statement

All data generated or analysed during this study are included in this article and its supplementary information files. link to the CARE Checklist and supplementary material: doi:10.6084/ m9.figshare. 28519463.

## Funding sources

This work was supported by Lanzhou Science and Technology Program Project(grant number: 2023-ZD-135).The fund is sponsored by the co-authors of this article.

## Ethics approval and consent to participate

This case report was conducted in accordance with the ethical standards of the institutional and/or national research committee. Written informed consent was obtained from the patient (or their legal guardian) for participation in this study and the use of their clinical data.

## Consent for publication

Informed consent was obtained from both the patient and their legal guardians for participation in the study and publication. This statement ensures compliance with ethical guidelines and confirms that consent for publication was obtained in writing. The details of the written informed consent are provided in the supplementary materials.

## Supplementary material

The following supplementary materials are available with this manuscript:

CARE Checklist: A checklist confirming adherence to the CARE guidelines for case report documentation. (Filename: CARE Checklist. docx)

Supplementary material-Table 1: We retrospectively analyzed previous CML patients with *BCR::ABL1* and Ph-negative status in blast crisis from database, such as PubMed, MEDLINE, Embase, Web of Science. And conducted a comprehensive review of the relevant literature. To date, only 39 cases of CML with Ph- development to AML have been reported globally, as shown in Table 1.(Filename: Supplementary material-Table 1.docx).

## CRediT authorship contribution statement

**Huan Liu:** Writing – review & editing, Writing – original draft, Data curation. **Yunxia Sun:** Writing – review & editing, Resources, Formal analysis. **Liangliang Li:** Validation, Investigation. **Yurong Zhang:** Validation, Investigation. **Yaxiong Zhou:** Validation, Investigation. **Pengyun Zeng:** Writing – review & editing, Conceptualization. **Lingling Yue:** Supervision, Project administration.

## Declaration of competing interest

The authors declare that they have no known competing financial interests or personal relationships that could have appeared to influence the work reported in this paper.

## Appendix Supplementary materials

Image, application 1
