# Supplementary material for: Rapid development of Philadelphia chromosome-negative AML in a CML patient with sustained major molecular response to tyrosine kinase inhibitor therapy
Source: Leuk Res Rep. 2025 Aug 21;24:100536. doi: 10.1016/j.lrr.2025.100536 (PMC12409312; doi:10.1016/j.lrr.2025.100536)
Supplement: Supplementary file 1 [file mmc1.docx]

**Supplementary material-table 1**

We retrospectively analyzed previous CML patients with *BCR::ABL1* and Ph-negative status in blast crisis from database and conducted a comprehensive review of the relevant literature. To date, only 39 cases of CML with Ph- progression to AML have been reported globally.

Table-1

| Case | Author  /Year | Age(yr)/Sex | Treatment for  CML | Best response  of cytogenetics | Best response  of molecular biology | Karyotype  /Gene mutations | Category in blast crisis | Interval  to AML  (month) | Treatment for  Ph- AML | Patient status at report |
| --- | --- | --- | --- | --- | --- | --- | --- | --- | --- | --- |
| 1 | Ohtsuka E /  1995 | 65/M | busulphan  IFN  Ubenimex | CCyR | NA | 48,XY,+8,+11 | AML | 51 | Ara-C | Dead |
| 2 | Fayad L/  1997 | 72/M | IFN | CHR | NA | 44,XY,del(1)(q32), del(5)(q13q34) | AML | 92 | MA(Mitozantrone+Ara-C) | Dead |
| 3 | R,Manley 1999 | 32/F | busulphan  IFN | MCyR | NA | 无 | AML | 288 | Palliative care | Dead |
| 4 | Chee et al  2003 | 42/M | HU, IFN | NR | NR | 复杂核型 | AML | 84 | HSCT,DLI, IM | Dead |
| 5 | Perel et al  2005 | 77/M | HU, IFN, IM | CCyR | MMR | 44,XY,del(5)(q13q31),der(6),t(6,17) (p25;q11.2),-7,17,der(20),t(7,20)(q11.2;p13), +mar | AML | 35 | Palliative care | Dead |
| 6 | Perel et al  2005 | 63/M | HU, IFN, IM | CCyR | NA | 45 XY,-7 | AML | 19 | FLAG | Dead |
| 7 | Craig Kovitz  2006 | 64/M | IFN,Ara-C, IM, NI | *PCyR* | Partial remission | 45, XY,-7 [4] | AML | 46 | IA(Idarubicin+Ara-C)  HSCT | Dead |
| 8 | Craig Kovitz  2006 | 51/F | IFN,IM | CCyR | MMR | -7 | MDS(RAEB-2) →AML | 65 | Azacitidine | Dead |
| 9 | Craig Kovitz  2006 | 55/M | IFN,Ara-C, IM | CCyR | MMR | 54,XY,+Y,+1,+4, +6, +8, +11, +14, +19, +2mar  46,XY,del(5)(q12) | MDS(RAEB-2) →AML | 71 | Salvage chemotherapy(NA) | Dead |
| 10 | Attaphol Pawarode  2007 | 42/F | IM | NA | NA | 无 | AML | 6 | HSCT | Dead |
| 11 | Elias Jabbour  2007年 | NA | IM | NA | NA | -7 | AML | NA | HSCT | Dead |
| 12 | Carmen, Fava  2008 | 70/M | IM  NA | CCyR | MMR | 47, XY, +13[14]  46, XY, t(2;14)(p11.2;q32) [1]  46, XY[1] | AML | 76 | NA | Dead |
| 13 | Pavel Dvorak  2009 | 41/F | HU,IFN,  Ara-C,IM | CCyR | NA | 47, XX,+8  inv(3)(q21q26) | AML | 120 | IA  HSCT | NA |
| 14 | Björn, Hackanson  2009年 | 74/M | HU,IFN,  Ara-C,IM | CCyR | NA | 46,XY, t(9;22) (q34; q11) [4] / 45，XY, -7 [4] / 46, XY [2] | MDS (RAEB-2)→AML | 192 | Decitabine | Dead |
| 15 | Kaori Karimata  2010 | 60/M | HU,IFN,IM | CCyR | MMR | 45,XX,-7[16] | MDS→AML | 120 | IM | Dead |
| 16 | Nina Larsson  2010 | 55/F | HU,IM,DA | MCyR | NA | 45，XX,- 7[17]/46，XX,t(9;22)[6]/46,XX[2] | AML | 40 | Proposed HSCT | Dead |
| 17 | Jad J. Wakim  2012 | 81/M | IFN,IM | CCyR | MMR | t(15;17)(q24;q21.1) | APL | 132 | ATRA+Idarubicin | Dead |
| 18 | Georgiou  2012 | 61/M | IM | CCyR | CMR | NPM1 | AML | 56 | IA(Idarubicin+Ara-C)  FLAG  Palliative care | Dead |
| 19 | Florence,Van Obbergh | 79/M | IM | *PCyR* | NR | del(5)(q21q34) [4]  del(20)(q12) [7]  MYC amplification | AML | 12 | Palliative care | Dead |
| 20 | Krysiak  2016 | 72/M | IM | CCyR | CMR | TET2, NPM1 PRPF8 | AML | NA | NA | NA |
| 21 | Krysiak  2016 | 54/M | IM | Lack of metaphases | CMR | NR2E1（TLX）missense mutation | AML | 60 | NA | NA |
| 22 | Carmelo Gurnari  2017 | 66/F | HU  splenectomy | NA | NA | i(X)(p10) | AML | 12 | NA | NA |
| 23 | Jiang JG,  2018 | 63/M | IM, DA | CCyR | MMR | 49-50,XY,+6,+8,del(11)(q21),+1–3mar[cp5]/46, XY[15].  MLL amplification | AML | 118 | NA | Dead |
| 24 | Matthew K. Stein  2018 | 70/M | IM | CCyR | CMR | 46,XY, del(7) (q22)  ATM, DNMT3A, IDH1, NPM1 | AML | 168 | IA(Idarubicin+Ara-C)  Palliative care | Dead |
| 25 | Gong Z  2019 | 48/M | NI, IM, DA,  BO, PO | CCyR | NA | 46,XY,t(11;17) (q23;q25) | AML | 31.9 | IA(Idarubicin+Ara-C)  clofarabine | Dead |
| 26 | Gong Z  2019 | 67/M | IM | CCyR | NA | 45, XY, -7  FLT3-ITD | AML | 38 | HU | Alive |
| 27 | Gong Z  2019 | 45/F | HU, NI, DA | CCyR | NA | KRAS,FLT3-ITD, FLT3-D835, WT1, PHF6, RUNX1 | AML | 72 | IA(Idarubicin+Ara-C)  cladribine +  midostaurin PO | Dead |
| 28 | Gong Z  2019 | 60/F | IFN,Ara-C, IM | CCyR | NA | NPM1 | AML | 187.2 | Decitabine | Dead |
| 29 | Gong Z  2019 | 78/F | IM, NI, BO | CCyR | NA | 47, XX,+8  SRSF2 | AML | 20.2 | Azacitidine  BO | Alive |
| 30 | Gong Z  2019 | 40/F | DA, PO | CCyR | NALost | None | AML | 82.1 | Lost | Lost |
| 31 | Huan Zhu  2019 | 56/F | IM，NI | CCyR | MMR | t(1；19) (p36;p11)[10]  CEBPA, ATRX, WT1, CSMD1, IKZF1, and LRP1B突变 | AML | 54 | IA(Idarubicin+Ara-C) | NA |
| 32 | Qian Jiang  2019 | 22/M | NI | CCyR | MMR | 46,XY,t(3;21)(q26.2;q22)[17]/46,XY[3] | MDS→AML | NA | Chemotherapy(NA) | Dead |
| 33 | Qian Jiang  2019 | 78/F | IM | CCyR | CMR | 45,XX,-7[19]/46,XX[1] | AML | NA | IM | Dead |
| 34 | Qian Jiang  2019 | 57/F | DA | CCyR | MMR | 45,XX,-7[19]/46,XX[1] | AML | NA | Chemotherapy(NA) | Lost |
| 35 | Qian Jiang  2019 | 28/F | NI | CCyR | MMR | 45,XX,-7[20] | AML | NA | Chemotherapy(NA) | NA |
| 36 | Qian Jiang  2019 | 49/M | NI | CCyR | Partial remission | 45,XX,-7[18]/45,idem,t(9;22)[2] | MDS→AML | NA | HSCT | Dead |
| 37 | Qian Jiang  2019 | 55/M | NI | CCyR | MMR | 47,XY,+8[4]/46,XY[18] | MDS→AML | NA | HSCT | Alive |
| 38 | Fumi Nakamura  2020 | 55/M | DA, BO | CCyR | MMR | NPM1 W287fs  IDH2 R140L | AML | 14 | DA(daunorubicin+Ara-C) | NA |
| 39 | Goranthala Bharathi Devi  2022 | 59/F | IM | CHR | MMR | 47, XX,+8 | AML | 42 | Decitabine  DA(daunorubicin+Ara-C) | NA |

Abbreviations: IFN interferon, *HU* hydroxyurea, *Ara-C* cytarabine, *IM* imatinib, *DA* dasatinib, *NI* nilotinib, *BO* bosutinib,*PO* ponatinib, *HSCT* hematopoietic stem cell transplantation, *DLI* donor lymphocyte infusion, *ATRA a*ll-trans retinoic ac*id, FLAG* fludarabine, Idarubicin, and granulocyte colony-stimulating factor, *CCyR* complete cytogenetic response*, MMR* major molecular response*,* *CHR* complete hematological remission,*CMR* complete molecular response, *MCyR* Major cytogenetic response, *PCyR* partial cytogenetic response, *NR* non-remission, *NA* not available.
